# Peruvian Maca (*Lepidium peruvianum*): (II) Phytochemical Profiles of Four Prime Maca Phenotypes Grown in Two Geographically-Distant Locations

**Published:** 2016-03

**Authors:** Henry O. Meissner, Alina Mscisz, Ewa Piatkowska, Marek Baraniak, Sebastian Mielcarek, Bogdan Kedzia, Elzbieta Holderna-Kedzia, Pawel Pisulewski

**Affiliations:** 1 Faculty of Health Studies, Charles Sturt University & Therapeutic Research, TTD International Pty Ltd, 39 Leopard Ave., Elanora, QLD 4221, Australia;; 2 Research Institute of Medicinal Plants, 27 Libelta St., 61-707 Poznan, Poland;; 3 Faculty of Food Technology, Cracow University of Agriculture, 122 Balicka St., 30-149 Krakow, Poland;; 4 Institute of Natural Fibres & Medicinal Plants, Wojska Polskiego 71-13, Poznan 30-630, Poland

**Keywords:** Four phenotypes, Glucosinolates, HPLC fingerprint, hypocotyl colours, Lepidium peruvianum, Maca

## Abstract

Peruvian Maca crops *(Lepidium peruvianum),* grown in two geographically-distant cultivation sites located at similar altitudes in the highlands of the Peruvian Andes (Junin at 4,200 m a.s.l. and Ancash 4,150 m a.s.l.), were used in the study. Four prime Maca phenotypes, distinguished by hypocotyl colours labelled as “Yellow”, “Purple”, “Red” and “Black” were selected to determine distribution in levels and corresponding ratios between individual Glucosinolates (Glucotropaeolin and m-methylglucotropaeolin) in an attempt to identify four Peruvian Maca phenotypes from analyses of powdered hypocotyls. There were highly significant differences (*P*<0.01) in hypocotyl weight/size of four Maca phenotypes harvested in two locations. The Junin crop represented a mostly “large” class (13.3 g) with “small” size hypocotyls (7.2 g), while a “small” class was predominant in Ancash (3.5 g). Powdered Yellow Maca showed significantly higher (*P*<0.001) microbial contamination than the other three, with Black Maca being the least infected. Only minor, statistically-confirmed differences were detected in nutritive characteristics between the four Maca phenotypes grown in Junin, however highly significant differences (*P*<0.01) in Glucosinolates existed between the Red and Black Maca grown in Junin and Ancash. Irrespective of the cultivation location, Red phenotypes showed the highest content of Total Glucosinolates, followed by Black and Purple, with the Yellow phenotype showing consistently lower levels. Highly significant *P*<0.01) differences determined in ratios of individual Glucosinolates between four Maca phenotypes grown in two locations, confirms an earlier assumption that sums of individual Glucosinolates, their ratios and profiles, may be feasible to explore in analytically identifying individual Maca phenotypes in pulverised marketed Maca products.

## INTRODUCTION

According to scientific classification, the genus *Lepidium* belongs to the tribe of Lepidieae and sections, Dileptium and Monoploca in the *Cruciferae (Brassicaceae)* family (1). It consists of approximately 175 species found worldwide (2), but only some of them are cultivated as crop plants of economic importance. Amongst them are well-known oilseeds (rapeseed), vegetables (cauliflower, cabbage, Brussel sprouts, garden rockets, water and garden cress, radish, etc.), spices (mustard) as well as fodders (fodder kale, fodder radish) to mention a few. Out of all known *Lepidium* species, Peruvian Maca - *Lepidium peruvianum* Chacon (*L. peruvianum*) is the only *Lepidium* species cultivated at high-altitude habitats in the Peruvian Andes (above 3,500 m up to 4500 m a.s.l.), where is known for its edible starch-containing subterranean part which forms an integral part of the locally-grown staple food. Natives of Peru living in the Andean highlands, traditionally supplement their daily meals with Maca in a variety of consumable dish forms, in the belief that it favorably affects energy, mood, fertility, improves sexual desire and decreases anxiety (3, 4). This plant, nearly extinct by the middle of the XX century was re-established in its native habitat as a cultivated cash crop, mostly due to research initiated in 1961 by Chacon Roldan (5). Chacon Roldan has provided a historically-based link of Peruvian Maca as a traditional medicinal plant cultivated on the Junin plateau in the highlands of the Peruvian Andes, to an ancient medicinal herb cultivated since the times of the Incas. It’s therapeutic, healing, preventative and energy stimulating properties have been recently confirmed by results obtained in the laboratory and in clinical research on human subjects (6-12).

Considered as a non-hormonal, adaptogenic herb, when taken regularly as a dietary supplement, Peruvian Maca exhibits hormone-regulating properties for wide range of age groups of men (4) and women (8, 9), helping to restore metabolic harmony and induce hormonal balance specific to gender and appropriate age-stage (3, 8, 9). Several groups of distinctive compounds have been detected in the Peruvian Maca hypocotyls, such as Alkaloids, Saponins, Tannins, Sugars, Anthocyanins, Starch, Fatty acids and aromatic Glucosinolates (13-16) - but none of them specifically – has yet been linked to the traditionally- and clinically-acknowledged therapeutic and medicinal properties of this plant (3, 4, 13, 17-19).

**Figure 1:**
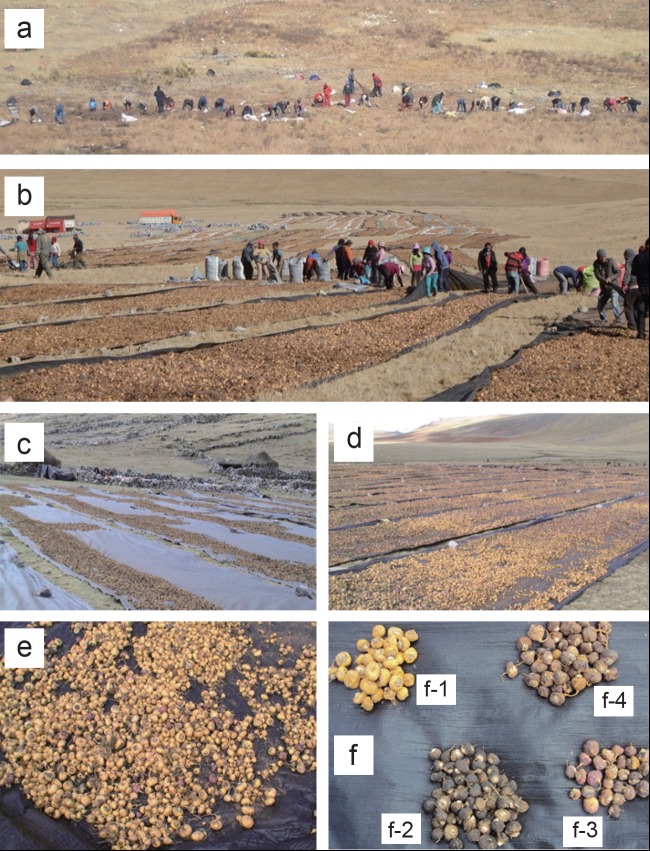
Traditional communal harvest of cultivated Peruvian Maca by natives of Andean highlands in Junin plateau [a], followed by “open-air drying” process of the harvested Maca crop [b, c], pre-selection [d] and separation of Maca phenotypes [e] according to four colours of Maca hypocotyls [f]: Yellow (f-1), Black (f-2), Purple (f-3) and Red (f-d) representing four prime phenotypes in mixed Peruvian Maca crop as used in this study.

Irrespective of the location of the cultivated sites in the Andean highlands, commonly harvested Maca crops (20, 21) are composed of differently-colored hypocotyls (17, 19), with Yellow, Red, Black and Purple (Figure 1) appearing to be dominant in the mixed crop (Table 1). Amongst several groups of biologically-active compounds reported in the Peruvian Maca hypocotyls (3-5, 13, 22, 23), high concentrations of aromatic Glucosinolates (benzyl and p-methoxbenzyl glucosinolates in particular) and their isothiocyanate derivatives have been linked to therapeutic and health properties of Maca (4, 14). In previous papers from this series (16, 24), Glucosinolates have been demonstrated as compounds which may help in identifying Maca as a unique Peruvian plant (24). Differently-colored Maca hypocotyls have shown different Glucosinolates in both concentrations (16, 23) and HPLC profiles. This could assist in identifying the individual Maca phenotypes (16).

**Table 1:** Morphologic Diversity of Maca Phenotypes - Percentage distribution of Maca hypocotyls originated in Junin (*Lepidium peruvianum* Chacon)

Colour of Hypocotyls (English and local *Spanish terms* used)	% Ref # 1	% Ref # 2	% Ref #3	Presence only (Ref #4)

Yellow - *Amarillo*	47.8	39	48	X
Red-White - *Rojo-Blanco*	16.5			
Purple-White - *Morado-Blanco*	9.0			
White-Red - *Blanco-Rojo*	6.3			
Grey - *Plomo*	5.4	2		X
Black - *Negro*	4.2	3	16	
Yellow-Red - *Rojo-Amarillo*	3.7			
Light (White) - *Blanco*	2.2			
Purple-White - *Blanco-Morado*	1.6			
Yellow-red - *Amarillo-Rojo*	1.3			
Grey-Light - *Plomo-Claro*	0.8			
Purple-Grey - *Morado-Plomo*	0.7			
Yellow-Light Grey - *Amarillo-Plomo-Claro*	0.5			
Purple - *Morado*		29	6	X
Yellow-Purple - *Amarillo-Morado*		13		
White-Purple - *Blanco-Morado*		12		
Red - *Rojo*			3	
*Higos*				X
*White*		2		
*Rosado cin Moradoen la Corona*				X
*Blanco con Lila*				X
*Lila con Blanco Jaspedo*				X
*Blanco Plomizo Humo*				X
*Blanco con Guindo an la Corona*				X
*Peco de Pito*				X
*Other colours*			27	

Ref #1: derived from 758 collections originating in Junin and Pasco District - Tello *et al*. (21) cited by L. Obregon, (17); Ref #2: derived from collections originating in Junin District - cited by C.F. Quiros & R.A. Cardenas (20); Ref #3: derived from 3 batches originating in Junin District - cited in previous study by Meissner *et al.* (6-9); Ref #4: derived from a commercial collection of Maca available for marketing (La Soberana, Lima, Peru); No percentage values given. Positions marked with “X” indicate presence of the particular colour according to the commercial terminology adopted by the Company.

Gonzales (19) has observed differences existing in the size and weight of hypocotyls derived from various Maca mixed crops. Therefore, it is reasonable to assume that, Maca crops cultivated in different habitats and composed of disparate percentage ratios between major phenotypes and sizes of hypocotyls, may influence their analytically-determined profiles of active compounds and, in particular, Glucosinolates (16).

In this paper, an attempt is made to present phenotypic distribution in crops of Peruvian Maca *(L. peruvianum)* cultivated in two geographically-distant locations in the Andean highlands (Junin and Ancash) labelled as “Yellow”, “Black”, “Red” and “Purple”. Simultaneously, nutritive values and corresponding phytochemical characteristics are determined on the Glucosinolate levels, as well we the ratios between the two major secondary metabolites measured in the four Maca phenotypes.

## MATERIAL AND METHODS

### Peruvian Maca (*L. peruvianum*)

The plant species was phyto-chemically characterized in one of the previous papers from this series (24).

Two Peruvian Maca crops, consisting of phenotype mixtures grown in two geographically-distant cultivation sites and located at similar altitudes in the highlands of the Peruvian Andes were used in the study. Four prime Peruvian Maca phenotypes, selected from the crops, representing an edible subterranean part of the plant, and distinguished by the color of hypocotyls labelled as “Yellow”, “Black”, “Red” and “Purple”, were chosen for laboratory studies.

Several commercial-size samples of hypocotyls representing four major Maca phenotypes were separated from mixed harvested Maca crops grown in two cultivation locations: the Junin plateau (9.53°S 77.53°W), situated at 4,200m a.s.l. and a newly-established “De Genaro” planting site situated in Ancash, at 4,150m a.s.l. on the western slopes of the Cordillera Blanca mountain range (11.48°S 74.98°W).

Both cultivation sites and the processing facility were attested for their organic status SKAL (25). Before being used in the study, samples of dried Maca hypocotyls from both locations were authenticated by Dr Gloria Chacon as representing cultivated Peruvian Maca *Lepidium peruvianum* Chacon and conforming to the characteristics representative of this plant species. (24).

At both cultivation sites, Maca hypocotyls were harvested according to the traditional system (Figure 1a), and dried for some 6 to 9 weeks until approximately 10% moisture content was reached. A Traditional “open air system” of dehydration was used within proximity of the cultivation sites, at high altitudes, on plastic mats with daily exposure to full sun-light and frequent mixing. (Figure 1b, 1c) This is the very system which is considered superior to that of the oven dried method currently used in commercial “modern” operations in Peru (Obregon, 2001 and Chacon 2003 – personal communications). During the process of drying, individual colored hypocotyls were pre-selected, and eventually separated (Figure 1d, 1e) into the four Maca phenotypes to be used in this study (Figure 1(f1-4)). A sample of Small class “fresh hypocotyls”, representing each of the four phenotypes currently being dried, was collected for Glucosinolate analyses within 24 hr from the harvest (in Junin location only).

Commercial batches of dried Maca hypocotyls selected from the Junin and Ancash cultivation sites, were packed into jute sacks, each weighing approximately 40 kg. These were then transported to a commercial operation in Lima (Peru) for further processing.

### Distribution of phenotypes in Maca crops

Distribution of a particular phenotype (identified by color) was assessed in two batches of mixed Maca crops originating in Junin and Ancash (9 MT and 6 MT respectively). This was performed by individually counting the basic colors of hypocotyls: Yellow, Black, Red and Purple in each of 12 randomly-selected 40 kg Maca lots from each crop. As soon as both batches had been delivered to the processing plant in Lima, they were manually separated into four distinctively colored phenotype groups. Other colors present in smaller percentage quantities, were bulked as remaining phenotypes.

Both the weight and number of the four colored groups of hypocotyls in each mixed Maca phenotype subsample lots were recorded. Comparison of the average individual hypocotyl weight (g/hypocotyl) was made by dividing the total weight (kg) by the number of counted hypocotyls within each phenotype group. The counted hypocotyls were expressed as a percentage (%) of the total number in each lot of mixed Maca crop. According to common local procedure, commercial lots of dry Maca delivered from the highlands are graded on size basis, with the “A” – large size (the highest price value) and “B” and “C” (small) considered of lesser value for the Maca processors. For the Maca crop originating in Junin, two grades were assessed – “A” (large) and “C” (small). The crop from the Ancash location only represented small sized (C) hypocotyls.

### Sample preparation

Dried Maca hypocotyls representing four phenotypes from each cultivation location, were cleaned (washing in water under pressure without any chemicals used in the process) and cut into chip-size pieces. Maca chips were then placed in a dehumidifier-dryer at 35oC to max 38oC until maximum 8% moisture was reached. The chips were then pulverized to particle size and passed through an 80 mesh size sieve. Powdered Maca phenotypes were packed into 5kg double wall plastic pouches formed with an oxygen and moisture barrier. These pouches were then sent for nutritional and biochemical analysis to Research Laboratories in Poland for further analyses.

### Analytical Procedures

**Nutritive characteristic:** The Official Method of Analysis of AOAC (26) was used to determine the levels of nutritive compounds: dry matter, total protein, raw fat, and total dietary fiber. The AOAC method numbers 934.06, 950.36, 935.38, 991.43 and 930.05 respectively were used. The content of total carbohydrate was calculated. Fatty acid compositions in the Maca samples were determined with the use of gas chromatography according to PN-EN ISO 5508:1996 (27) on Shimadzu QP5050A (Shimadzu, Kyoto, Japan) equipped with a column SP-2560 (100 m × 0.25 mm × 0.25 μm, Supelco, Bellefonte, Pennsylvania, USA).

**Glucosinolate analysis using HPLC procedure:** The Maca hypocotyls collected from processed 12 lots of the four Peruvian Maca phenotypes: Yellow, Black, Red and Purple originating in Junin and Ancash, and pulverized to 80 mesh size, were analyzed for Glucosinolates according to the method by Li *et al.* (28), together with the protocol and modifications described previously (16). Three 1g samples from each phenotype were extracted in methanol, followed by purification using a solid-state extraction method and Glucosinolate detection at 235nm against Glucotropaeolin (Benzylglucosinolate), Sinigrin (2-propenyl glucosinolate derived from black mustard) and m-methoxyglucotropaeolin, as external reference standards for Glucosinolates.

The data was presented as mean ± SD (n=2). One-way, parametric analysis of variance (Statistica v. 8.1, StatSoft, Inc., Tulsa, OK, USA) was applied for testing the differences between experimental treatments. Duncan’s Test was used for the identification of statistically significant differences at a level of *p*<0.05.

**Bacterial contamination in four Peruvian Maca phenotypes:** Differences in the susceptibility of Peruvian Maca phenotypes to bacterial contamination was tested on the 12 lots (approx. 40kg each), of commercially batched, mixed-colored Maca hypocotyls harvested in Junin. From each lot, four Maca phenotypes were selected prior to processing. This was followed by the collection of 5 kg samples of each Maca phenotype from bulk powdered product at their packaging stage. Standard techniques for detection of the degree of microbiological contamination was adopted after Kedzia (29), originally-developed for use in testing contamination in herbal material and herbal supplements. Microbiological concentration data is presented as Total Viable Bacterial Count (TVC) and on the log10 scale (as is general microbiological practice). One-way Analysis of Variance (ANOVA) was used on the principle basis by Moore and McCabe (30), that more than two groups are tested for differences in means and there is no direct relationship between the experimental units in the groups.

## RESULTS

### Morphological diversity in four Phenotypes of Peruvian Maca crops (*L. peruvianum*) harvested in Junin & Ancash

Maca hypocotyls from two geographically distant Andean plateaus, Junin and Ancash, were classified according to their weight/size, with distinction made between the four main phenotypes, together with expressing participation of the counted colored hypocotyls as a percentage of the mixed Maca crops (Table 2). According to the hypocotyl weight/size, two classes of Maca were determined from the Junin crop: Large-class A (>10 g) and Small-class B (<10 g), while in Ancash one Small-class C was recorded only (<5 g).

**Table 2:** Morphologic diversity of Phenotypes in Peruvian Maca (*L. peruvianum*). Distribution of dried Maca hypocotyls in commercial crops of mixed phenotypes originating in Junin (sizes large & small) and Ancash (small size only) assessed on g/hypocotyl basis (with corresponding +/- SD) and number of differently-colored expressed as a percentage of the total counted hypocotyls in the mixed Maca crop (% - *in italics*)* and two reference values used for comparison only (not included in statistic evaluation)

Colour of Hypocotyls	Large Junin^1^ g^5^ (%)	Small Junin^1^ g (%)	Small Ancash^2^ g (%)	Ref. 3 Ninacaca g	Ref. 5 Junin (%)

Yellow (*Amarillo)*	13.2 +/- 1.21 ab I *(51.5%) B*	6.9 +/- 0.47 a II *(55.0%) B*	3.5 +/- 0.31 a III (*61.1%) A*	10.4	(48)
Purple (*Morado)*	13.0 +/- 1.14 ab I *(22.8%) A*	8.4 +/- 0.57 b II *(18.4%) A*	3.4 +/- 0.38 a III *(22.2%) A*	n.d.^6^	(6)
Red (*Rojo)*	14.8 +/- 1.33 a I *(15.3%) A*	6.8 +/- 0.51 a II *(20.2%) B*	3.7 +/- 0.32 a III *(9.9%) C*	9.2	(3)
Black (Negro)	12.6 +/- 1.07 b I *(10.4%) A*	6.6 +/- 0.49 a II *(6.3%) B*	3.4 +/- 0.41 a III *(6.8%) B*	10.7	(16)
Average weight (g)	13.3 I	7.2 II	3.5 III	10.1	n.d.^6^

* Values with unlike lower case letters within the columns indicate an existence of differences in hypocotyls’ weight (g) between Yellow, Purple, Red and Black Peruvian Maca phenotypes at statistically significant level (*P*<0.05), while unlike Roman numbers within the rows (within each phenotype) indicate significant differences (at *P*<0.01) in weight (g) of dried hypocotyls obtained from Junin (Large and Small) and Ancash (Small only).

1 derived from 12 commercial sub-samples originating in Junin District, consisting of both Large (“A”) and Small size (“C”) hypocotyls (2008 crop);

2 derived from 12 commercial sub-samples originating in Junin District representing Small size (“C”) hypocotyls only - (2008 crop);

3 derived from collection of Maca in Ninacaca, Pasco District (2006 crop) cited in study by Gonzales et al (2006) (No percentage values given);

4 derived from 3 collections originating in Junin District - cited in previous study by Meissner et al. (2005, 2006). Percentages only, weight of individual hypocotyls was not determined at that time.

5 Average weight of hypocotyls (g) and Standard Error of Mean (+/- SEM);

6 n.d. - not determined.

It appears that the Junin hypocotyls grew significantly (*P*<0.01) larger, with the average Large class weighing 13.3g and Small 7.2 g, while the Ancash crop in the same harvest year reached 3.5 g only with no hypocotyls larger than 5 g detected in the Maca batches from Ancash.

In comparing the colors of hypocotyls, Red phenotype showed significantly (*P*<0.05) higher weight in the Junin ‘Large class’, while in the Junin ‘Small class’, Purple hypocotyls showed significantly (*P*>0.05) heavier hypocotyls as compared to other three phenotypes within their respective classes in Junin. There were no significant differences (>0.05) observed in hypocotyls’ weight between the four Maca phenotypes ‘Small class’ harvested in Ancash.

When comparing percentage distribution of hypocotyl colors within the same class and location, it appears that Yellow hypocotyls represented more than 50% of mixed hypocotyls in batches of Maca, with a significantly (*P*<0.05) higher proportion of Yellow hypocotyls (61.1%) recorded in the Small class of crop grown in Ancash when compared to the Small class of hypocotyls harvested in Junin (55%).

Purple color hypocotyls in the Junin ‘Small class’ mixed crop were in significantly (*P*<0.05) higher proportions when compared to the ‘Large class’ from the same location. Identic color in the Ancash ‘Small class’ showed a highly significant lower participation (*P*<0.01) than in the Junin ‘Small class’ with a significantly lower (*P*<0.05) participation when compared to that observed in the Junin ‘Large class’. Percentage participation of Black-colored hypocotyls in the Junin ‘Large class’ was significantly higher (*P*<0.05) when compared to the ‘Small class’ Black hypocotyls obtained from Junin and Ancash.

### Nutritive Characteristics of Four Maca Phenotypes

There were significant differences detected (*P*<0.05) in individual components of the proximate nutritional composition of four Maca Phenotypes (Table 3). The most distinctive and significant differences (*P*<0.05) existed between Black Maca and other three phenotypes, where levels of Crude Protein, Crude Fat and Crude Ash in Black hypocotyls were significantly (*P*<0.05) lower than in other three colors of hypocotyls. A simultaneous statistically (*P*<0.05) higher level of Carbohydrates and Fiber existed in Black Maca phenotype, when compared to the other three hypocotyl colors. The highest levels of Crude Protein were observed in Yellow and Red phenotypes, while Crude Fat and Ash showed the highest values in Purple phenotypes. The lowest level of Carbohydrate was observed in Purple phenotypes, with Fiber levels the in Red phenotypes.

**Table 3:** Nutritional composition of commercially-processed pulverised four Peruvian Maca (*L. peruvianum*) phenotypes distinguished by the colour of hypocotyls: Yellow, Purple Red and Black cultivated in Junin plateau^1^

Phenotype (pulverised Maca hypocotyls)	Dry Matter g/100g	Crude Protein (Nx6.25) g/100g	Crude Fat g/100g	Ash g/100g	Carbo-hydrates g/100g	Fibreg/100g

Yellow	93.2 ± 0.2^b,c^	14.4 ± 0.0^b^	0.9 ± 0.0^b^	4.8 ± 0.0^a^	79.9 ± 1.0^a,b^	10.3 ± 0.1^a,b^
Purple	93.8 ± 0.4^c^	14.1 ± 0.0^b^	1.6 ± 0.0^c^	5.1 ± 0.0^c^	79.2 ± 0.2^a^	10.6 ± 0.0^b,c^
Red	91.3 ± 0.1^a^	14.4 ± 0.2^b^	1.0 ± 0.1^b^	5.0 ± 0.0^b^	79.7 ± 0.3^a,b^	10.1 ± 0.2^a^
Black	92.8 ± 0.4^b^	13.5 ± 0.2^a^	0.8 ± 0.0^a^	4.7 ± 0.0^a^	81.0 ± 0.3^b^	10.7 ± 0.1^c^

1 Mean values shown in columns and denoted by different letters are statistically significant (*p*<0.05).

There were no distinctive differences found (no statistic evaluation available) in profiles of Fatty Acids determined in the four Maca Phenotypes (Table 4) except C18:1 chain (E)-octadec-9-enoic (elaidic). This Fatty Acid was found in Yellow phenotypes (19.85%) to be substantially – and nearly, 100% higher than the concentrations of this Fatty Acid detected in Purple, Red and Black Maca (within the range 10.1% to 10.3%).

**Table 4:** Profiles of Fatty Acids in the four phenotypes of commercially-processed pulverised cultivated Peruvian Maca (*L. peruvianum*) distinguished by the colour of hypocotyls: Yellow, Black, Red & Purple cultivated in Junin plateau^1^

	Fatty Acid	Yellow	Purple	Black	Red

C14:0	Tetradecanoic (myristic)	0.22	0.19	0.15	0.2
C15:0	Pentadecanoic	0.18	0.18	0.16	0.18
C16:0	Hexadeconic (palmitic)	21.15	21.37	21.31	20.99
C16:1	9-cis-Hexadecenoic (palmitoleic)	0.55	0.52	0.46	0.52
C17:0	Heptadecanoic (margaric)	0.36	0.34	0.34	0.33
C18:0	Octadecanoic (stearic)	2.23	2.03	2.25	2.09
C18:1	(E)-octadec-9-enoic (elaidic)	19.85	10.14	10.35	10.21
C18:2	9, 12-octadecadienoic (linoleic)	36.69	35.44	37.2	35.26
C20:0	Icosanoic (arachidic)	0.39	0.35	0.33	0.29
C18:3	9,12,15 Octadecatrienoic (α-Linoleic)	22.9	24.35	22.66	25.18
C22:0	Docosanoic (behenic)	0.3	0.28	0.25	0.26
C24:0	Tetracosanoic (lignoceric)	0.2	0.24	0.25	0.2
C24:1	(Z)-Tetracos-15-enoic (nervonic)	0.36	0.33	0.38	0.35

1 Single determination only.

### Glucosinolate analysis using HPLC procedure

Total Glucosinolate concentrations in fresh and dry hypocotyls ‘Small class’ of Maca collected from Junin, and dry ‘Small class’ hypocotyls from the Ancash location, are summarized in Table 5. While Total Glucosinolates analyzed in “fresh” Yellow, Purple and Red hypocotyls were significantly (*P*<0.05) higher than in dried hypocotyls from the same location (Junin), there was no such statistically confirmed difference between fresh and dry Black phenotype hypocotyls (*P*>0.05). Red Maca phenotypes showed the highest content of Total Glucosinolates in both fresh (analyzed at the initial stages of drying when still fresh), and dry hypocotyls. The determined values were confirmed to be statistically higher (*P*<0.05) when compared to the other three phenotypes of Maca cultivated in Junin.

**Table 5:** Total Glucosinolate concentration (g%) in four phenotypes of Peruvian Maca (L. peruvianum) hypocotyls Small class: Yellow, Purple, Red and Black collected in Junin location at the altitude 4,200 m a.s.l. and analysed^1^ as fresh or after being dried with simultaneous comparison to dried four Maca phenotypes Small class as harvested in Ancash location (at 4,150 m a.s.l.)*

Maca Phenotype	Fresh hypocotyls Junin (Small)	Dry hypocotyls Junin (Small)	Dry hypocotyls Ancash (Small)	± SD

Yellow (*Amarillo)*	0.973 a I	0.218 a II	0.175 a II	± 0.322
Purple (*Morado)*	0.821 a I	0.303 ac II	0.300 b II	± 0.217
Red (*Rojo)*	1.821 b I	0.508 b II	0.220 ab III	± 0.274
Black (Negro)	0.460 c I	0.406 bc I	0.289 b II	± 0.101
Mixture of individualvPhenotypes	n.d.^2^	0.316 ac I 3	0.302 b I 4	± 0.076

* Values with unlike lower case letters within the columns indicate an existence of differences between Yellow, Purple, Red and Black Peruvian Maca phenotypes at statistically significant level (P<0.05), while unlike Roman numbers within the rows indicate significant differences (P<0.05) between results obtained from hypocotyls analysed as fresh or after being dried in Junin and Ancash location.

1 Total Glucosinolates results obtained with the use of the method by Li *et al.* (2001) and determined as the sum of Glucotropaeolin and m-methoxyglucotropaeolin, used as external standards;

2 n.d. - not determined;

3 Average weight of hypocotyls in the Maca Small class harvested in Junin = 7.2g; Percentage distribution of hypocotyls (by numbers) in analysed Junin Small class: Yellow/Purple/Red/Black = 55/18.4/ 20.2/6.3 (%);

4 Average weight of hypocotyls in the Maca Small class harvested in Junin = 3.5g; Percentage distribution of hypocotyls (by numbers) in analysed Ancash Small class: Yellow/Purple/Red/Black = 61.1/22.2/ 9.9/6.8 (%).

When comparing the content of Glucosinolates in four phenotypes which originated in Junin and Ancash, the ‘Small class’ of hypocotyls from Junin showed a significantly (*P*<0.05) higher concentrations in the Red and Black hypocotyls. Similar differences in the Yellow and Purple phenotypes were not statistically confirmed (*P*>0.05). The Red and Black hypocotyls from the dry Maca crops originating in Junin, contained a significantly (*P*<0.05) higher Glucosinolate concentration as compared to the Yellow and Purple phenotypes. On the other hand, the dry Purple hypocotyls from Ancash showed the highest concentration of Total Glucosinolates, the values of which were statistically lower (*P*<0.05) than in the Yellow and Black phenotypes. There were no statistically-confirmed differences (*P*>0.05) detected in the values of Total Glucosinolates recorded within the mixed, dry phenotypes collected in Junin and Ancash.

**Figure 2:**
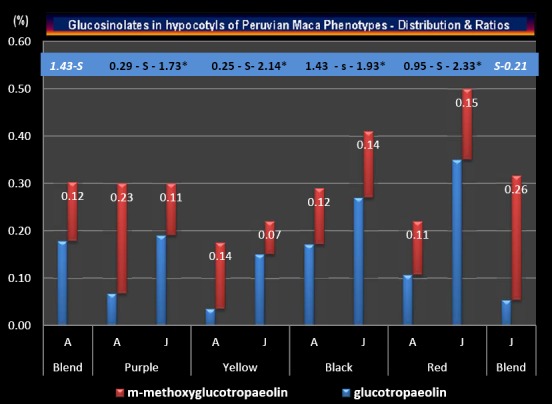
Total Glucosinolates in four commercial batches of cultivated Peruvian Maca (L. peruvianum) phenotypes distinguished by the colour of hypocotyls: Purple, Yellow, Black and Red and distribution patterns in their two secondary metabolites: Glucotropaeolin (GLTRP) and m-methoxyglucotropaeolin (MEGLT) showing calculated concentration ratios (values in the highlighted row above each set of bars with results corresponding to each phenotype) harvested in Ancash (A) and Junin (J). For comparison, the results from analysis of typical blends of Maca hypocotyls processed as mixed crops harvested in Ancash (A Blend) and Junin (J Blend) are represented by bars at the extreme left and right of the diagram with the letter -S- referring to significant difference in ratios of individual Glucosinolates existing in the two locations A and J (corresponding figures marked by white bold text). *Capital letter (– S -) between A and J bars indicate statistically highly significant difference in GLTRP/MEGLT ratio values within each Maca phenotype (P<0.01), while lower case letter (– s -) denotes significant difference existing between the ratio values at P<0.05 level.

Distribution and Ratios of individual Glucosinolates determined in the ‘Small classes" of hypocotyls which represent the four Peruvian Maca phenotypes cultivated in Junin and Ancash, are summarized in Figure 2. While there was no statistical differences within Total Glucosinolate content between the blend of hypocotyls harvested in Junin and Ancash (Table 5), there were highly significant differences (*P*<0.01) in ratios of Glucotropaeolin (GLTRP) and m-methoxyglucotropaeolin (MEGLT) recorded in Ancash (1.43) and in Junin (0.21). However, GLTRP/MEGLT ratios determined within individual phenotypes grown in Ancash, ranged from 0.25 through 0.29 to 0.95 for Yellow, Purple and Red phenotype respectively, while for the same phenotypes grown in Junin, the ratios were 1.73, 2.14 and 2.33 respectively. The differences between GLTRP/MEGLT ratios recorded for Yellow, Purple and Red Maca phenotypes cultivated in Ancash and Junin were statistically, highly significant, at *P*<0.01 level (denoted in Figure 2 by capital –S- letter), while differences in the same ratio for Black Maca grown in the two locations, was less distinctive, but still significant at P<0.05 level (denoted in Figure 2 by lower case –s- letter).

### Microbial contamination of hypocotyls in four Peruvian Maca phenotypes

The total viable microbial contamination (TVC) in representative samples of powdered hypocotyls collected from commercial batches of the four processed Maca phenotypes originating in Junin is summarized in Table 6. Of the all four Maca phenotypes, Yellow hypocotyls showed the highest level of contamination with Gram-positive aerobic *bacillus* strains only (2.2 × 10^4^ cfu/g). While there were no detectable or low microbial contaminants (occasional Gram-positive cocci strain colonies only) detected in the Black Maca phenotypes (<1 × 10^1^ cfu/g), the Purple Maca showed moderate contamination (1.1 × 10^3^ cfu/g) with Gram-negative rod-shaped bacteria (*Bacilli*) with samples of the Red Maca hypocotyls showing the presence of both Gam-positive aerobic *bacillus* strains and Gram-positive *cocci* strains (2.0 × 10^2^ cfu/g).

**Table 6:** Degree of bacterial contamination expressed as Total Viable Count (cfu/g) and as log10 cfu/g, as well as strains of bacteria detected in four Peruvian Maca phenotypes (*L. peruvianum*) Yellow, Purple, Red and Black selected from a commercial batch of mixed-coloured dried Maca hypocotyls harvested in Junin location at the altitude 4,200 m a.s.l. and delivered for processing to the Lima factory prior to sampling at the packaging stage

Maca Phenotype	Mean Total Viable Count (TVC)^1^ (cfu/g)	TVC^1^ as percentage of Yellow Phenotype =100%	(TVC)^1^ (log10 cfu/g)*	SD (log10)	Strains of Bacteria detected

**Yellow (*Amarillo)***	2.2 × 10^4^	100	4.33 a	0.151	Gram-positive aerobic *bacillus* strains only
**Purple (*Morado)***	1.1 × 10^3^	5.09	3.04 b	0.665	Gram-negative rod-shaped bacteria (*Bacilli*)
**Red (*Rojo)***	2.0 × 10^2^	0.93	2.30 b	0.513	Gram-positive aerobic *bacillus* strains & Gram-positive *cocci* strains
**Black (Negro)**	<1 × 10^1^	0.01	0.60 c	0.577	Gram-positive cocci strain
**Detected difference**	(*P*<0.001)	(*P*<0.001)	(*P*<0.001)		

* TVC (log10 cfu/g) values with unlike lower case letters within the columns indicate an existence of differences in a degree of microbial contamination between Yellow, Purple, Red and Black Peruvian Maca phenotypes at statistically highly significant level (*P*<0.001).

1 TVC (Total Viable Bacterial Count) in the sample bulked from 12 commercial sub-samples originating in Junin District, consisting of Large size (“A”) hypocotyls (n=3).

Since microbiological data are frequently *right skewed* (31), statistical analysis of data related to microbial contamination of four Maca phenotypes was conducted after log10 transformation of the cfu/g results. Statistically significant differences (*P*<0.001) existed in the level of mean TVC of the four Maca phenotypes expressed as Log10 cfu/g. The differences were generally in the order of 1 to 3 log10 cfu/g (Table 6).

## DISCUSSION

### Morphological diversity of Phenotypes in Peruvian Maca crops (*L. peruvianum*) harvested in Junin & Ancash

Distribution of the four phenotypes into two hypocotyl blends derived from bulk batches of mixed Maca crops, harvested from two geographically-distant, organically-certified locations in Junin and Ancash, was made on the basis of weight/size which categorized classes of Maca hypocotyls marketed in Peru and commonly termed as “Large” and “Small”. Apparently, farmers and Maca dealers in Peru charge more for larger class of hypocotyls (class A – large) as opposed to smaller size hypocotyls class B and C, which carry lower price tags.

Percentage distribution of the four main phenotypes in two Peruvian Maca crops grown at similar altitudes in Junin and Ancash (Table 2) showed, that irrespective of the weight/size Class of hypocotyls, the Yellow phenotype was dominant within the mixed Maca crop (51.5% to 61.1%). The above results were in agreement with the observation made previously by other authors (17, 20, 21) who all reported that Yellow phenotypes were dominant within Peruvian Maca crops (Table 1). As well, the results align with the Maca crops previously used by the author in a series of clinical studies on Maca (6-9).

Quiros & Cardenas (20) stated that purple phenotypes are the second prime color in the Maca crop, which historically was identified in the original habitat of cultivated Maca. However, Obregon (17) quoting data obtained by Tello *et al.* (21) determined Red-White as the second dominant color in the Maca crop. Back in 1992, Tello *at al.* (21) analyzed 758 samples-batches of Maca hypocotyls originating from different locations in the Department of Junin, and separated some 13 different phenotypes distinguished by characteristic colors of hypocotyls (Table 1) with some 48% representing yellow , 16% red-white, 9% purple-white and 4% black, with other hypocotyl colors participating in a lesser quantities.

Contrary to the percentage participation mentioned earlier (20, 21), Gonzales *et al*. (19) reported differences existing in ratios between average weight and distribution of individual Maca phenotypes harvested in three regions of Andean highlands, with the Black hypocotyls harvested in the Junin district showing substantially heavier weights than Red and Yellow phenotypes. The results reported in this paper confirmed Gonzales *et al.* (19) observations in the ‘Large Class’ of hypocotyls which originated from the Junin crop, where Red phenotypes were heavier than the other in this Class, while purple hypocotyls were heavier in the ‘Small Class’ of hypocotyls harvested in both the Junin and Ancash locations. It must be underlined, that both the Large and Small class of hypocotyls harvested in Junin, were statistically (*P*>0.05) heavier (13.3 g and 7.2 g respectively) than all the phenotypes harvested in Ancash (3.5 g). This indicates that the weight/size of hypocotyls may be significantly influenced by the environmental, soil, cultivation practices and other factors peculiar to the location where Maca is grown.

### Composition of the four phenotypes in Junin-grown Maca crop

Comparison of overall chemical composition of the basic phenotypes sampled from the mixed Maca crop grown in Junin, showed that the differences, although some statistically-confirmed as significant, were not distinctive enough to help in identifying individual phenotype on the basis of its basic chemical analysis. Back in 1994, Dini *et al.* (32), presented in-depth chemical composition of Peruvian Maca samples, however without any reference to the phenotype/color of the samples used in their work. Gutierrez (33), who studied nutritive values of three Maca phenotypes Yellow, Red and Black have not found distinctive enough differences in chemical compositions to distinguish different Maca phenotypes. Similarly, Villanueva (34) observed some differences in protein fractions (albumins, glutelins and prolamin) of two “Light” and “Dark” colored Maca hypocotyls, and concluded that the differences were not sufficiently distinctive to allow for identification of color in analyzed Maca samples from the analysis of protein fractions.

There are a number of compounds identified within Maca hypocotyls (5, 14, 35), but no specific one has been shown to be distinctive enough to be used for identifying Maca phenotypes. One distinctive class of secondary metabolites which have been found only in Maca, have been termed, the Macamides (36-39). This class of metabolites has been identified as n-benzylhexadecanamide, n-benzyl-(9Z)-octadecenamide, n-benzyl-(9Z, 12Z)-octadecadienamide, n-benzyl-(9Z, 12Z, 15Z)-octadecatrienamide and n-benzyloctadecanamide. Dry Maca hypocotyls contain Macamides (determined as benzylated alkamides: N-benzyl-5-oxo-6E, 8E-octadecadienamide and N-benzylhexadecanamide) within the range from 0.0016% to 0.0123% (37). Although they may identify the product representing or containing Maca, has not been suggested as the method to be used in identifying Maca phenotypes. There is neither sufficient research data nor clinical evidence that Macamides, as the class of secondary metabolites or any specific form on its own, could be directly linked to the therapeutic effects of Maca on human organism and its gender-selective physiological action.

Clemente *et al.* (23) who analyzed the concentrations of major secondary metabolites in hypocotyls and the leaves of Maca in a controlled planting experiment in the Peruvian Andes, at 4130 m above sea level found, that the color type of Maca hypocotyls significantly affected most secondary metabolites, with the exception of β-sitosterol and campesterol. The grey-colored, yellow and violet Maca hypocotyls were rich in glucosinolates, Macaene and Macamides respectively, and the hypocotyls were richer in those compounds than leaves. They (23) concluded that the color of Maca hypocotyls has to be considered in production of Maca preparations, as it is associated with variations in concentrations of distinct bioactive metabolites – the fact which has been clinically-confirmed by Gonzales (4).

In previous papers from this series (16, 24), it has been shown that the profiles of the group of compounds – Glucosinolates and their intermediary metabolites, may help in the identification of individual Maca phenotypes selected from mixed Maca crops and in commercial, pulverized Maca products. Also an attempt has been made to use DNA profiles in identifying Maca phenotypes so as to distinguish Yellow Maca from the two other phenotypes studied (16). The previous work on the application of analytical techniques to identify Maca phenotypes from Glucosinolate profiles, confirmed earlier stipulation by Johns (14), Li *et al.* (28) and Gonzales *et al.* (4), who considered Glucosinolates as the group of compounds which reflect physiological functionality and influence therapeutic potential of cultivated Maca blends and Maca phenotypes, used individually as dietary supplements (19).

In this work, an attempt has been made to find and select laboratory techniques, which could allow for distinction of phenotypical differences characteristic to the individual colors of Maca hypocotyls in pulverized material - a problem, which has not been satisfactorily resolved in any laboratory work to date. This would allow for assurance of the quality of marketed commercial Maca preparations, thus verifying their chemically determined phenotypical identity and corresponding biological activity, responsible for specific physiologic functionality in men and women.

### Glucosinolates concentration and distribution ratios

Maca hypocotyls contain relatively higher levels of glucosinolates when compared to other cruciferous crops like white cabbage and cauliflower (28). Therefore, it has been assumed that Glucosinolates – as a group of characteristic compounds, may potentially be used as a marker when screening Maca for its peculiar functionality and gender-related physiological effects (4).

Results from screening Peruvian Maca cultivars, presented in one of previous papers from this series (16), may confirm the above assumptions, since studied Maca phenotypes exhibited differences in both concentrations and HPLC profiles. Significant reductions in Total Glucosinolates concentration were observed in this study, after drying fresh hypocotyls of Yellow, Purple and Red Maca phenotypes. This is in line with observation made by Yabar *et al.* (40), who linked reduction in Glucosinolates level during the process of drying to myrosinase activity in the cells of fresh Maca hypocotyls affected by the process of drying. Reduction in Total Glucosinolates after drying fresh hypocotyls as reported in this paper is consistent with observations by Jing Li *et al.* (41), who reported some 57% decrease in Glucosinolate content after raw Maca was dried for 24 hr.

Results from this study indicate that although there were significant differences in Total Glucosinolates between individual Maca phenotypes, and between locations where Maca was grown (particularly in Red and Black Maca phenotypes), the Total Glucosinolate result was not a reliable measure to identify phenotypes of Maca grown in the same and/or in different locations. Piacente (35), who studied the secondary metabolites in the Maca tuber, found that the methanolic extract, in addition to free sugars and amino acids, contained uridine, malic acid and its benzoyl derivative also contained the two Glucosinolates identified as glucotropaeolin (GLTRP) and m-methoxyglucotropaeolin (MEGLT). These two Glucosinolates were also reported in previous studies (16), as distinctively characterizing HPLC resolution profiles in three Maca phenotypes: Black, Red and Yellow.

While there was no difference in the Total Glucosinolates content within the mixed Maca crop harvested in Junin and Ancash, when individual Glucosinolates identified on HPLC chart as Glucotropaeolin and m-methoxyglucotropaeolin were used in calculating individually ratios in their concentrations for each location (Figure 2), there was a significant difference in GLTRP/MEGLT ratio for the crop harvested in Junin (0.21) and Ancash (1.43). However, the same ratios for each individual phenotype grown in Junin were significantly higher as compared to values of the GLTRP/MEGLT ratios recorded for Maca phenotypes grown in Ancash. This may indicate that the calculated higher GLTRP/MEGLT ratios recorded in Junin may be attributed to the location, or it could be the result of heavier/larger hypocotyls determined by the weight/size of the “Small” Class grown in Junin (average 7.2 g) as compared to Ancash (3.5 g).

The interpretation of the GLTRP/MEGLT ratio – although interesting from its sensitivity viewpoint – not observed in Total Glucosinolates measurements, would need to be investigated further as a potential tool in identifying – if not individual Maca phenotypes – then, as a potential indication of biological potency of preparations derived from different Maca hypocotyls harvested from different planting sites. Before hypothesizing further, this assumption would need to be tested in bioassays and in laboratory models.

Clement *et al.* (23) reported that colours of Maca hypocotyls are associated with variations in concentrations of distinct bioactive metabolites and Glucosinolates in particular as well as to a lesser degree to Macaene and Macamides contents. The same authors (23) were also referring to each Maca colour as having different biological effects which they stipulated is linked to distinctive differences in profiles of bioactive metabolites. Different biological effects of Black, Red and Yellow Maca has been demonstrated in series of study by Gonzales and his Research Group (4), showing that phytochemical variability existing in Peruvian Maca phenotypes, identified by the color of hypocotyls, may be linked to different physiological responses of men and women ingesting specific Maca phenotype or their blends. Different responses to ingestion of differently-colored Maca hypocotyls were confirmed in clinical studies by Gonzales (19) on men and Meissner *et al.* (8, 9) on pre- and post-menopausal women. However, while physiological effects of individual phenotypes of Peruvian Maca (Red or Black) used in clinical trials on male subjects were consistent from trial to trial (10-12), systematic review of results on the use of mixed Maca phenotypes in women, based on searching 17 databases by Li *et al.* (42), found no such consistency.

Using the Cochrane ‘risk of bias’ assessment tool, Maca-based experimental intervention was compared to a placebo for the treatment of menopausal symptoms in healthy perimenopausal, early postmenopausal, and late postmenopausal women, provided inconclusive result (42). This could be attributed to reported in this paper and also observed by Gonzales (19), differences in size/weight of hypocotyls derived from mixed Maca crops cultivated in different locations. Inconsistent results from the use of dietary supplements marketed by different suppliers and manufacturers may be the result of different Maca crops from which Maca products are derived and may also be attributed to variation in proportions of differently-colored hypocotyls of Maca (individual Maca phenotypes), which exist in crops grown and harvested in different Maca plantations located in different cultivation regions and locations as shown in Table 1.

### Microbial Contamination of hypocotyls in four Peruvian Maca phenotypes

The mathematical treatment of microbiology data as presented in Table 6 was done for two reasons. Firstly, microbiological data is frequently *right skewed* (31). This is due to the exponential growth nature of microorganisms and it is natural to observe some samples with very high microbial counts. This violates the assumptions of the statistical analysis and inflates the mean and standard deviations calculated from the data. Consequently, the statistical assumptions are better met after log10 transformation. Secondly, microbiologists are familiar with log10 transformed data. In the context of comparing different samples, starting with the most contaminated sample, a reduction of 1 log10 is equivalent to a 90% lower microbial count and a 2 log10 reduction in values indicates lower contamination equivalent to a 99% reduction in reference to the sample chosen as the reference or arbitrary selected baseline. Furthermore, microbiologists generally accept a 1 log10 difference between two treatments as practically significant (30).

### Factors which may influence observed differences in Maca crops grown in Junin and Ancash

There is not an easy task to provide interpretation for an existence of substantial and highly significant differences in phenotypic characteristics, size/weight of hypocotyls, complex biochemical composition with Glucosinolates and GLTRP/MEGLT ratio in particular as detected in four Maca phenotypes cultivated in Junin and Ancash. Maca crops in both locations were sawn, grown and harvested in similar manners in terms of centuries-old traditional agro-cultivation practices (except heavy tractors being used in ploughing the soil prior to sawing instead of manual field preparation). The seeds used for cultivation in Ancash were obtained from the same plantation in Junin from which Maca crop was used in the comparison study presented in this paper. Therefore, the origin of seeds and agronomic factors may be eliminated as the reason for the existing substantial difference in size of hypocotyls observed in the two cultivation locations and corresponding biochemical composition. In addition, environmental influences – which seemed to be similar and peculiar in the two high Andean plateaus at the altitude of about 2,200m a.s.l. may point out at possible differences in soil characteristics which may be an important factor contributing to differences in the size of the Maca crops, weight and biochemistry of hypocotyls harvested in Junin and Ancash.

Analysis and characteristics of soil samples taken from two positions in each field where Maca was grown – as presented in Table 7, indicate an existence of substantial differences in soil pH, with Ancash soils being more neutral as compared to Junin, where soils were uniformly acidic. Lead and Zink were in higher concentrations in soil samples collected in Ancash as compared to Junin. This was associated with overall higher Cation concentration in Ancash soil (Ca^+2^ and Mg^+2^ in particular), while soils in Junin showed much higher contents of Al^+3^ + H^+^. Also Junin soils had higher mud and clay fraction as compared to Ancash. Thus, more pronounced growth of Maca hypocotyls in Junin soils may be explained by more favourite soil conditions leading to better uptake of minerals by Maca plants cultivated on a soil with higher clay fraction. This aspect of improved mineral uptake from Peruvian type soils on higher plant growth was demonstrated by Oelsligle *et al* (43), who, using exchangeable soil Potassium model, demonstrated an existence of high correlation between growth of the plant biomass the presence and apparent degree of crystallinity of illite in the clay fraction of the soils. The aspect of soil characteristic in plateaus of Andean highlands where Maca is cultivated and its possible effect on resultant yields of Maca crops and their therapeutic functionality measured by proven biochemical indices warrants further study.

**Table 7:** Soil characteristic* in two geographically-distant Maca cultivation locations: Junin and Ancash

Soil Component	Unit	Junin 1	Junin 2	Ancash 1	Ancash 2

pH		4.19	4.74	7.72	5.9
CaCO_3_	%	0	0	0	0
P	ppm	5.6	13.7	15.2	28.2
K	ppm	61	241	154	256
Pb	ppm	16.87	15.87	27.15	29.33
Zn	ppm	29.25	113.25	269.92	173.17
**Cations (Sum)**	meq/100 g	**3.67**	**6.19**	**31.08**	**25.21**
Ca^+2^	meq/100 g	0.97	3.04	28.5	22.7
Mg^+2^	meq/100 g	0.3	0.45	2.2	1.67
K^+^	meq/100 g	0.19	0.72	0.31	0.58
Na^+^	meq/100 g	0.1	0.08	0.07	0.06
Al^+3^ + H^+^	meq/100 g	2.1	1.9	0	0.2
**Soil Fractions**					
Sand/grit	%	58	56	n/d**	75
Mud	%	32	28	n/d	20
clay	%	10	16	n/d	5

* Soil Analysis conducted at the Laboratorio de Analisis de Suelos, Planta s, Aguas y Fertilisantes, Universidad Nacional Agraria la Molina, Lima, Peru;

** n/d - not determined.

Variation in sizes of hypocotyls used in the production of a specific marketable Maca preparation with clinically proven functionality may render the product delivering inconsistent physiological outcome due to differing proportions between outer layer (skin) of the hypocotyl and its core region in the small and large class of hypocotyls. As opposed to large class, in smaller hypocotyls, as derived from geometry of hypocotyls, one may expect a higher participation of the outer layer of hypocotyls – the cortex, in relation to the bulk starchy core. Therefore, it may be assumed, that the pulverized product from the smaller hypocotyls would contain higher concentration of antocianines (responsible for the external color of the hypocotyls) and, possibly, other biologically-active Maca compounds, which, usually tend to be located in/and close under the skin. In the large class of hypocotyls, a starchy core provides a prime bulk of processed Maca product, which nutritionally may be superior, but again may show lower biological activity as compared to the small class. This obviously may not be valid for Maca and needs to be analytically tested. However, an assumption has been made that, for the final product with consistent biological and therapeutic activity, it is essential to maintain standardized phenotype ratio of known size hypocotyls in order to eliminate possible variations in biological potency of the product used by the end user for well-determined purposes. It may be assumed further, that different phenotypical composition of the Maca crop, as well as the size of Maca hypocotyls received from different plantations for the processing and production of specific Maca preparations could excerpt different resultant physiological effects.

## CONCLUSIONS

There were highly significant differences (*P*<0.01) in hypocotyl weight/size between the four Maca phenotypes harvested in the two cultivation locations, with the Junin crop representing mostly a “Large” class (13.3 g) and “Small” size hypocotyls (7.2 g), while a substantially smaller hypocotyl class was predominant in Ancash (3.5 g).

While only minor statistically-confirmed differences in nutritive characteristics were detected between the four phenotypes grown in Junin, distinctive, highly significant differences (*P*<0.01) existed in Total Glucosinolates, detected only within the Red and Black Maca grown in Junin and Ancash.

Irrespective of the cultivation location, Red phenotypes displays the highest content of Total Glucosinolates, followed by Black and Purple with the Yellow phenotypes consistently showing the lowest levels.

A comparison of phenotypic distribution and corresponding Glucosinolates levels were measured as Glucotropaeolin and m-methylglucotropaeolin. Highly significant *P*<0.01) differences were determined in ratios of individual Glucosinolates in the four Maca phenotypes grown in two locations. This confirms earlier assumptions that the content of individual Glucosinolates, both sums and their ratios, may be feasible to explore in analytically identifying individual Maca phenotypes, thus helping in verifying the identity of marketed Maca products. The above observation warrants further laboratory and practical testing.

Yellow Maca samples showed significantly higher (*P*<0.001) microbial contamination than the other three, with Black Maca sample being least infected.

## ABBREVIATIONS

AOAC: Official Methods of Analysis
a.s.l.: Above sea level
C14 – C24: Chain length of 14 to 24 Carbons
cfu/g: Microbial contamination: counted colonies per g material
DNA: deoxyribonucleic acid
g: Gram
GLTRP: Glucotropaeolin
HPLC: High Performance Liquid Chromatograph
hr: Hour
H_2_O: Distilled water
ID: Identity; Identifier of a unique object
*L. peruvianum*: *Lepidium peruvianum* Chacon
Log_10_: Microbial count converted to log_10_ format
M: Mole
m: Meter
mm: Millimetres
MEGLT: m-methoxyglucotropaeolin
Mix: Mixture
ml: Millilitres
MT: Metric Tonne
PMP: Peruvian Maca Project
*P*<0.01: Statistically important difference at *P*<0.01 level
*P*<0.05: Statistically important difference at *P*<0.05 level
Ref: Reference
SD: Standard Deviation
TVC: Total Viable Bacterial Count
USA: United States of America
v/v: On volume/volume basis
°S: Degrees South
°W: Degrees West
μ: Micron
μl: Microliter
μm: Micrometer
%: Percentage
°C: Degrees of Celsius
<: Less than
>: More than
